# Influence of Post-Processing on S-Phase Formation During Plasma Nitriding of Additively Manufactured Inconel 939

**DOI:** 10.3390/ma19010130

**Published:** 2025-12-30

**Authors:** Piotr Maj, Joanna Radziejewska, Ryszard Diduszko, Michał Marczak, Rafał Nowicki, Podolak-Lejtas Anna, Tomasz Borowski, Ryszard Sitek

**Affiliations:** 1 Faculty of Mathematical & Nature Sciences, School of Exact Sciences, Cardinal Stefan Wyszynski University, Kazimierza Wóycickiego 1/3. 21, 01-938 Warsaw, Poland; 2 Institute of Manufacturing Technology, Faculty of Mechanical and Industrial Technology, Warsaw University of Technology, Narbutta 85, 02-524 Warsaw, Poland; joanna.radziejewska@pw.edu.pl (J.R.); michal.marczak@pw.edu.pl (M.M.); rafal.nowicki1@pw.edu.pl (R.N.); anna.lejtas@pw.edu.pl (P.-L.A.); 3 Polish Academy of Science, Institute of Physics, Al. Lotników 32/46, 02-668 Warsaw, Poland; ryszard.diduszko@ncbj.gov.pl; 4 National Centre for Nuclear Research, ul. Andrzeja Sołtana 7/3, 05-400 Otwock-Swierk, Poland; 5 Faculty of Materials Science and Engineering, Warsaw University of Technology, Wołoska 141, 02-507 Warsaw, Poland; tomasz.borowski@pw.edu.pl (T.B.); ryszard.sitek@pw.edu.pl (R.S.)

**Keywords:** Inconel 939, plasma nitriding, S-phase, expanded austenite, additive manufacturing, surface treatment

## Abstract

Active screen plasma nitriding (ASPN) of additively manufactured nickel-based superalloys represents an understudied surface enhancement pathway. This study presents the first systematic investigation of ASPN applied to additively manufactured Inconel 939 (IN 939), evaluating four distinct post-processing routes combining heat treatment atmospheres (argon versus air cooling), vibratory finishing, and lapping under identical nitriding parameters (450 °C, 8 h, 25% N_2_ + 75% H_2_, 3 hPa). Contrasting nitriding behaviours emerged as a function of the post-processing route: the air-cooled thermal treatment (HT-air-vibr-lap) promotes formation of a thick Al/Cr-rich oxide layer (10–15 µm) that substantially inhibits nitrogen diffusion, resulting in thin and discontinuous nitrided layers. Conversely, the inert atmosphere route (HT-Ar-vibr-lap) circumvents oxide formation, enabling continuous S-phase (expanded austenite, γN) layer development of a 6.4 ± 0.3 µm thickness with exceptional surface hardness (~1200 HV, representing 3–4× enhancement relative to base material). X-ray diffraction confirmed S-phase formation with refined lattice parameter (3.609 Å) and secondary nitride phases (CrN-type and NbN/TaN-type precipitates). The post-processing sequence—particularly heat treatment atmosphere and mechanical finishing methodology—emerged as a critical controlling parameter for S-phase formation efficiency and mechanical properties of nitrided layers in additively manufactured nickel-based superalloys. This work addresses a knowledge gap distinct from the existing literature on conventional Inconel systems, establishing that controlled surface modification through post-processing can achieve the required properties.

## 1. Introduction

Additive manufacturing (AM), notably Laser Powder Bed Fusion (LPBF), has revolutionised the production of complex parts from high-performance alloys like Inconel 939. This nickel-based superalloy is widely used in the aerospace industry, for example, in turbine blades and vanes, due to its high strength and resistance to creep and oxidation at elevated temperatures [1,2]. However, parts made with LPBF often have unique surface and microstructural features. While these can offer opportunities, they also create challenges for surface treatment. LPBF enables the production of shapes that are very difficult or impossible to achieve with conventional methods, but it often results in high surface roughness (Ra~10–25 μm) [3,4]. The layer-by-layer process, partial melting of the powder, and rapid cooling rates [5] result in surfaces with unmelted particles, “stair-step” effects, and pores. These features significantly impact the performance of subsequent treatments, such as nitriding.

Nitriding is a thermochemical process in which nitrogen diffuses into the surface of a metal, forming hard nitrides and inducing compressive stresses. It improves surface hardness, wear resistance, and fatigue life [6,7]. For nickel-based superalloys, nitriding is usually performed at 500–650 °C [8,9]. Numerous studies have demonstrated positive results for the nitriding of conventional Inconel alloys. For example, Smith et al. [10] reported that plasma nitriding increased the hardness of Inconel 718 from 350 HV to 850 HV. At the same time, Johnson and Williams [11] showed that gas nitriding of Inconel 625 improved both corrosion resistance and mechanical properties. For AM alloys, the situation is more complex. The rough surfaces of as-built parts can lead to non-uniform nitrogen diffusion [12,13]. Additionally, AM microstructures with dendritic growth, porosity, and residual stresses can impact both diffusion and nitride formation [14,15].

Due to these challenges, several studies emphasise the importance of post-processing before nitriding. Zhang et al. [16] showed that rough surfaces (*Ra* > 15 μm) reduce coating uniformity and adhesion. Heat treatment is also critical. Because LPBF involves high-speed cooling (10^3^–10^6^ K/s), it produces non-equilibrium structures with high dislocation density and segregation [17,18]. Heat treatment can homogenise the microstructure, relieve stresses, and control precipitation of strengthening phases [19,20]. For Inconel 939, conventional heat treatment involves solution treatment at 1160–1180 °C, followed by ageing at 850–900 °C [21,22]. However, AM-made Inconel may require different parameters due to its distinct microstructure [23].

Another important step is improving the surface finish. Machining AM parts is often impractical because of their complex shapes, thin walls, or internal channels. Vibratory finishing has emerged as an effective alternative. This process utilises abrasive media in a vibrating bowl to reduce roughness and smooth surfaces [24,25]. Chen et al. [26] reduced the roughness of LPBF Ti-6Al-4V parts from *Ra* = 12.5 μm to *Ra* = 0.8 μm using vibratory finishing. The process also induced compressive stresses, improving fatigue resistance [27]. Effectiveness depends on factors such as media type, time, and part geometry [28]. For nickel-based alloys, vibratory finishing with metallic media can improve hardness and residual stresses through work hardening [29,30,31]. However, the effect of subsequent heat treatments on this hardened layer must be carefully considered [32].

Inconel 939 (IN 939) is widely recognised as a key material for high-temperature turbine and aerospace applications due to its exceptional creep resistance, fatigue strength, and thermal stability [33,34,35]. Recent systematic reviews highlight significant progress in the additive manufacturing (AM) of IN 939 via laser powder bed fusion (LPBF), demonstrating that AM-processed components can achieve mechanical properties comparable to, or in some cases surpassing, those of conventionally cast materials [33,35]. Nevertheless, LPBF processing intrinsically induces surface irregularities, residual stresses, and defect populations that may limit mechanical performance and restrict direct use in critical parts [33,35]. Consequently, post-processing—encompassing heat treatment, mechanical finishing, and thermochemical surface engineering—plays a central role in unlocking the full performance potential of AM IN 939 [33,35,36,37].

Although several studies have investigated heat treatment strategies for AM IN 939, showing that the cooling atmosphere significantly affects microstructural evolution and residual-stress development [37,38,39], the downstream influence of these conditions on nitriding behaviour remains insufficiently characterised. This knowledge gap is particularly relevant because the nitriding response of nickel-based superalloys is highly sensitive to the subsurface state, including defect density, dislocation structure, and surface topography—features directly shaped by the preceding post-processing steps. While plasma nitriding has been successfully applied to alloys such as Inconel 718 and 625, systematic evaluations of active screen plasma nitriding (ASPN) specifically tailored to LPBF-produced IN 939 remain scarce [35,40,41]. As a result, the combined effects of heat treatment atmosphere, mechanical finishing, and thermochemical processing have not yet been comprehensively assessed for this alloy.

To address this gap, the present study provides the first integrated assessment of how different post-processing sequences—including variations in heat treatment atmosphere, vibratory finishing, and lapping—affect S-phase formation and the resulting mechanical and tribological performance during ASPN of LPBF-fabricated IN 939. Despite the growing body of literature on AM post-processing and nitriding, existing studies typically consider these operations in isolation, and only a limited number focus specifically on LPBF-produced IN 939 [33]. Key open questions include the influence of cooling rate and atmosphere on the reactivity of the surface layer, the impact of mechanical finishing on heat-treated surfaces, the combined effects of sequential treatments on nitrogen transport and nitride-layer architecture, and the identification of processing routes that maximise nitriding efficiency.

The present work addresses these issues through a systematic comparison of multiple post-processing configurations (e.g., HT-Ar-vibr-lap, HT-air-vibr-lap), with a particular focus on the interplay between heat treatment, surface conditioning, and ASPN outcomes. The study investigates how these sequences influence S-phase development, nitrogen incorporation, hardness, layer adhesion, and frictional behaviour, enabling the identification of post-processing routes that deliver both enhanced surface hardness and improved functional durability. By providing the first comprehensive benchmark of integrated post-processing strategies for AM IN 939, this work fills a critical gap in the current state of the art, which remains largely focused on Inconel 718/625 and conventionally manufactured superalloys.

## 2. Materials and Methods

### 2.1. Workpiece

The samples were fabricated from Inconel 939, a nickel-based superalloy with the chemical composition presented in Table 1.

The specimens were manufactured using an EOS M 100 metal 3D printer (EOS GmbH, Krailling, Germany). Gas-atomised Inconel 939 powder with a particle size range of 15–40 µm was used. The LPBF process was conducted with the following parameters: layer thickness, 20 µm; hatch distance, 50 µm; stripe pattern width, 5 mm with a 0.1 mm overlap; and layer rotation, 67°. The applied volumetric energy density was E = 75 J/mm^3^.

### 2.2. Heat Treatment

Post-processing heat treatment was performed according to the manufacturer’s recommendations (BIBUS HOLDING AG, Fehraltorf, Switzerland). The procedure consisted of:

- Solution annealing at 1374 K for 4 h, followed by rapid air cooling;
- Secondary annealing at 1273 K for 6 h;
- Final annealing at 1073 K for 4 h in a stirred argon atmosphere.

Two heat treatment variants were applied: (1) rapid air cooling after solution annealing (HT-air), and (2) slow cooling in argon atmosphere after secondary annealing (HT-Ar).

### 2.3. Vibratory Finishing

After LPBF and heat treatment, vibratory surface finishing was performed using a custom-designed station developed for small tensile specimens. The process was conducted for 4 h at a rotational speed of 1800 rpm, with an RMS vibration amplitude of 1.4 mm; steel balls with a diameter of 6 mm served as the abrasive medium.

### 2.4. Nitriding Treatment

The active screen plasma nitriding (ASPN) process was carried out at a temperature of 450 °C (723 K) for eight hours in a gaseous atmosphere composed of 25% N_2_ and 75% H_2_, under a pressure of 3 hPa. The treatment was performed using an active, perforated screen made of AISI 304 steel to enhance the diffusion of nitrogen into the surface layer and to ensure a uniform nitrided layer formation without edge effects across all sample variants. The process was conducted via a semi-industrial device produced by the Institute of Precision Mechanics in Warsaw, Poland. The nitriding was performed for both Ar and Air heat-treated samples.

### 2.5. Microstructural Characterisation

The microstructure was examined on cross-sections prepared perpendicular to the building direction. Samples were cut, mounted, ground, polished, and etched using standard metallographic procedures. Observations were performed with an optical microscope and a Keyence VK-X100 confocal laser scanning microscope. For selected specimens, chemical analysis was conducted using Energy Dispersive X-ray Spectroscopy (EDS).

### 2.6. Surface Characterisation

Surface texture was characterised using a Keyence VK-X100 confocal microscope (Keyence Corporation, Osaka, Japan) and a Form Talysurf stylus profilometer (Taylor Hobson, Leicester, England) equipped with a 2 µm radius tip. Measurements were performed in accordance with ISO standards [31]. Surface topography was evaluated on an area of 1 × 1 mm, and the following stereometric parameters were analysed: *Sa*, *Sp*, *Sz*, and *Ssk*. Roughness was assessed for samples in four conditions: as-built (LPBF), LPBF + heat treatment in air/argon, LPBF + abrasive post-processing, and after nitriding.

### 2.7. Mechanical Properties of Nitrided Layers

The nanomechanical properties were determined using a NanoTest Vantage system (Micro Materials Ltd., Wrexham, Wales, UK). Nanoindentation tests were performed using Berkovich diamond indenter under a maximum load of 30 mN. The loading and unloading rates were 120 mN/min, with a 10 s dwell time at maximum load. Five indents were performed per sample cross-section. Elastic modulus and hardness values were calculated from displacement curves using the Oliver and Pharr method [42].

Microhardness measurements were performed with a Vickers indenter under a load of 5 g. Indents were performed on cross-sections at varying depths to determine hardness profiles across the nitrided layer.

### 2.8. Adhesion Testing

The adhesion of the nitride layers was evaluated using scratch testing with an MCT module (Anton Paar, Graz, Styria, Austria). A Rockwell C diamond indenter with a 200 µm radius was employed. Progressive load tests were conducted with a load range of 0.03 N to 30 N, a scratch length of 3 mm, and a speed of 6 mm/min. Acoustic emission and friction force signals were continuously recorded during testing. Pre- and post-test scans were performed at a constant load of 0.03 N. Surface panoramas of the scratches were acquired using a confocal microscope.

### 2.9. XRD Analysis

Diffraction patterns were acquired using a divergent X-ray beam and Bragg–Brentano parafocusing geometry on a Bruker D8 Advance diffractometer equipped with a θ/θ goniometer (radius 280 mm). The X-ray tube was fitted with a Cu anode, operated at 40 kV and 40 mA (λCuKα_1_ = 1.5406 Å). The scattered radiation was collected using a LYNXEYE XE-T detector operating in high-energy-resolution mode (ΔE < 380 eV at 8 keV, with an energy window optimised for CuKα lines) and in 1D acquisition mode without Ni filtering. The detector matrix consisted of 192 strips (each 0.075 mm wide), covering 2.941° of 2θ. The primary optics included a 0.5 mm fixed divergence slit and 2.5° axial Soller slits, while the detector optics contained only 2.5° axial Soller slits. Measurements were performed in the 2θ range of 20–120°, with a step size of 0.01° and a counting time of 1 s per step.

Phase identification was carried out using the Bruker DIFFRAC.EVA V6 software with the ICDD PDF-4+ 2024 database [43].

Quantitative phase analysis and structural refinement were performed using DIFFRAC.TOPAS software. The refinements were conducted using the fundamental parameters profile fitting (FPPF) method to account for instrumental contributions, combined with the Rietveld refinement approach [44]. Microstructural parameters, such as lattice constants, crystallite size, and microstrain, were optimised using the double-Voigt function approach, ensuring accurate modelling of size and strain broadening effects, as well as background profile fitting.

## 3. Results

### 3.1. Microstructure

**Figure 1:**
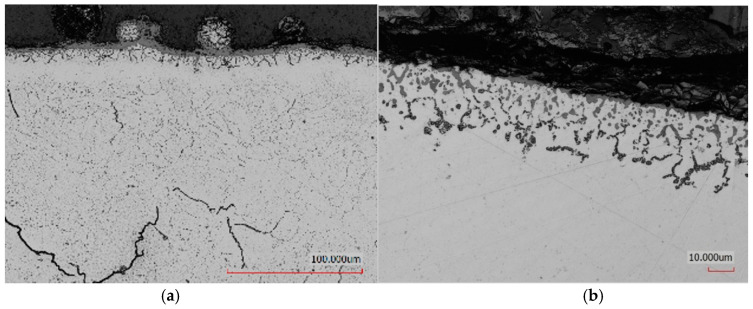
Cross-sectional microstructure of LPBF-manufactured Inconel 939 after post-processing: (**a**) heat treatment in air (HT-air) showing continuous oxide layer formation in subsurface region; (**b**) heat treatment in argon followed by vibratory finishing (HT-Ar-vibr) demonstrating reduced oxide layer thickness. Optical microscopy, unetched sections. Scale bar: 20 µm.

Figure 1 presents the subsurface microstructure of samples produced via additive manufacturing (3D printing) followed by distinct post-processing treatments. Figure 1a corresponds to samples subjected to heat treatment with air cooling (HT-air), while Figure 1b shows those treated with argon cooling and subsequent vibration-assisted machining (HT-Ar-vibr).

Following 3D printing and heat treatment, the microstructure exhibits a critical characteristic distribution of defects: isolated near-surface cracks occur rarely (near-surface region remains largely defect-free), while a network of microcracks is systematically identified in the central core region, with no porosity detected in either location. This defect distribution—concentrated microcracks in the core rather than near the surface—reflects the thermal history of the LPBF sample: the surface undergoes rapid cooling (10^5^ K/s), generating moderate residual stresses that are partially relieved through plastic deformation without crack formation. In contrast, the core material experiences slower cooling through successive layer redeposition cycles, creating steeper stress gradients and higher triaxial stress states that exceed the local fracture toughness, resulting in the observed microcrack network. Importantly, the absence of porosity combined with the presence of microcracks indicates that cracking is stress-driven (mechanical) rather than gas-porosity-driven (processing defect).

In the case of HT-air-treated samples, a continuous oxide layer approximately a few micrometres thick formed on the surface. This oxidation extended into the subsurface region to a depth of 10–15 µm and was also present on residual powder particles adhering to the surface which acted as an effective barrier against nitrogen diffusion, resulting in a thin and locally discontinuous nitrided layer. In contrast, although abrasive machining reduced the thickness of this oxide layer, oxygen-enriched regions (appearing dark in Figure 1b) remained near the surface. Conversely, samples heat-treated in an argon atmosphere (HT-Ar) did not exhibit a thick oxide layer, though a thin oxide film was still present on the surface after heat treatment. The HT-Ar-vibr-lap samples showed no such impediment, allowing the formation of a uniform nitrided layer approximately 6.4 ± 0.3 µm thick.

**Figure 2:**
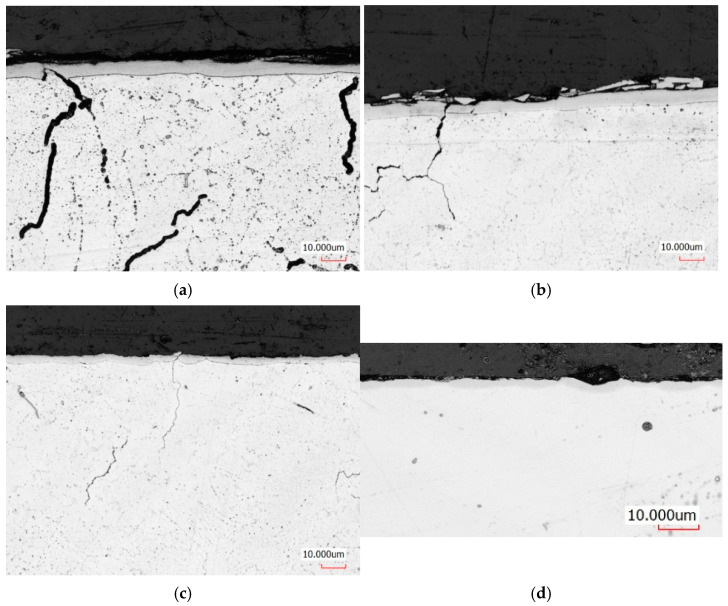
Subsurface microstructure of LPBF-manufactured Inconel 939 after plasma nitriding (450 °C, 8 h) with different post-processing sequences: (**a**) HT-air-vibr-lap (heat treatment in air, vibratory finishing, lapping); (**b**) HT-Ar-vibr-lap (heat treatment in argon, vibratory finishing, lapping); (**c**) HT-Ar-lap (heat treatment in argon, lapping only); (**d**) HT-Ar-vibr (heat treatment in argon, vibratory finishing without lapping). Nitrided layer thickness varies from 3.0 µm (**d**) to 6.4 ± 0.3 µm (**a**,**b**), showing expanded austenite (S-phase) formation. Light optical microscopy, etched sections. Scale bar: 5 µm.

Figure 2 presents the subsurface microstructure of additively manufactured IN 939 nickel-based alloy samples following ASPN process at 450 °C, applied after various post-processing conditions.

All samples developed a distinct surface-modified zone after nitriding, though the thickness and morphology of this layer varied significantly depending on the preceding surface preparation sequence. The sample that underwent only heat treatment and lapping (Figure 2c) exhibited the thinnest nitrided layer, approximately 3.8 ± 0.3 µm. In contrast, specimens processed with both vibro-abrasive finishing and subsequent lapping (Figure 2a,b) formed a substantially thicker and more uniform layer, averaging 6.4 µm. When vibro-abrasive treatment was applied without the final lapping stage (Figure 2d), the nitrided layer became locally discontinuous, reaching a maximum thickness of approximately 3 µm.

Microstructural analysis reveals that, regardless of the post-processing route, the nitrided layers are generally continuous and uniform through their entire thickness. The later XRD patterns and the micrographs confirm the formation of the expanded austenite phase (S-phase, γ_N_), characteristic of low-temperature nitriding processes. A slightly reduced thickness of the nitrided layers in the samples heat-treated in an argon atmosphere (Figure 2b,d) suggests the possible formation of a thin oxide film on their outer surfaces. This oxide layer—if not entirely removed during pre-nitriding polishing—may have acted as a diffusion barrier, locally limiting nitrogen penetration into the substrate and thereby decreasing nitriding efficiency.

A detailed examination of the near-surface region also revealed microcracks within the substrate, oriented perpendicular to the growth direction of the nitrided layer. These cracks remained unfilled during the process and may negatively influence the local mechanical integrity and corrosion resistance of the material.

### 3.2. Chemical Composition and Elemental Distribution of Nitrided Layers

**Figure 3:**
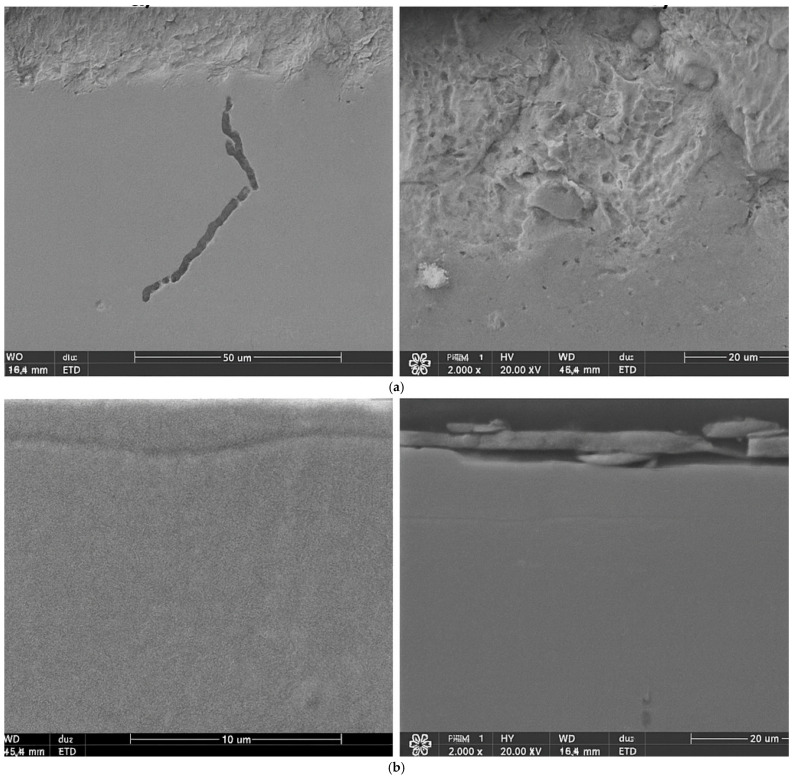

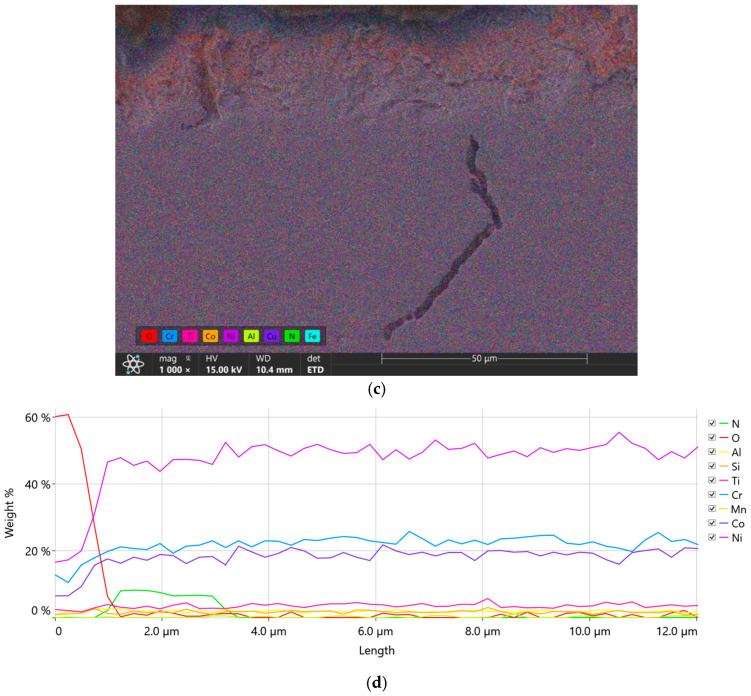
Energy-dispersive X-ray spectroscopy (EDS) analysis of nitrided layers: (**a**) SEM micrograph of HT-air-vibr-lap sample showing an inhomogeneous layer with porosity; (**b**) SEM micrograph of HT-Ar-vibr-lap sample showing a uniform, dense nitrided layer; (**c**) elemental composition mapping revealing nitrogen (N)—green, oxygen (O)—red, chromium (Cr)—blue, Nickel (Ni)—purple and aluminium (Al)—yellow distributions across the nitrided layer-substrate interface. Air-cooled samples show elevated oxygen and reduced nitrogen content compared to argon-cooled variants—scale bar: 2 µm. (**d**) Linescan starting from the surface with respectable element content with matching colors.

The chemical composition analysis of the nitrided layers revealed distinct differences depending on the preceding heat and surface treatment processes. Figure 3 presents representative cross-sectional microstructures with the marked X-ray microanalysis points. In the case of the HT-air-vibr-lap sample (heat treatment in air, followed by vibro-abrasive finishing and lapping), the nitrided layer exhibited local inhomogeneity and noticeable porosity (Figure 3a,b). In contrast, specimens heat-treated in argon—HT-Ar-vibr-lap and HT-Ar-lap—displayed a more uniform and compact morphology. However, the HT-Ar-lap sample (without vibro-abrasive finishing) showed local interfacial delamination between the layer and the substrate (Figure 3d).

EDS elemental maps (Figure 3c) confirmed that the HT-air-vibr-lap layer contained a significantly higher oxygen concentration compared to the argon-treated specimens. This indicates partial oxidation of the surface during heat treatment in air and subsequent limited nitrogen uptake during the nitriding process. The elevated oxygen and aluminium contents observed in this layer are consistent with the formation of an Al–O-enriched oxide film, which likely hindered nitrogen diffusion. In this sample, nitrogen was detected only in trace amounts, confirming a suppressed nitriding effect.

In contrast, both HT-Ar-vibr-lap and HT-Ar-lap samples showed explicit nitrogen incorporation, with concentrations of approximately 15 at.% and 10–16 at.%, respectively, indicating effective nitrogen diffusion under the applied process parameters. The similar chemical composition of the substrate matrix, recorded at a distance of ~12 µm from the surface in all analysed samples, confirms that the diffusion zone was limited to the near-surface region and that neither nitrogen nor oxygen was detected beyond this depth.

These findings demonstrate that the atmosphere used during heat treatment and the efficiency of surface cleaning before nitriding play a crucial role in determining the chemical composition and uniformity of the resulting nitrided layer.

**Figure 4:**
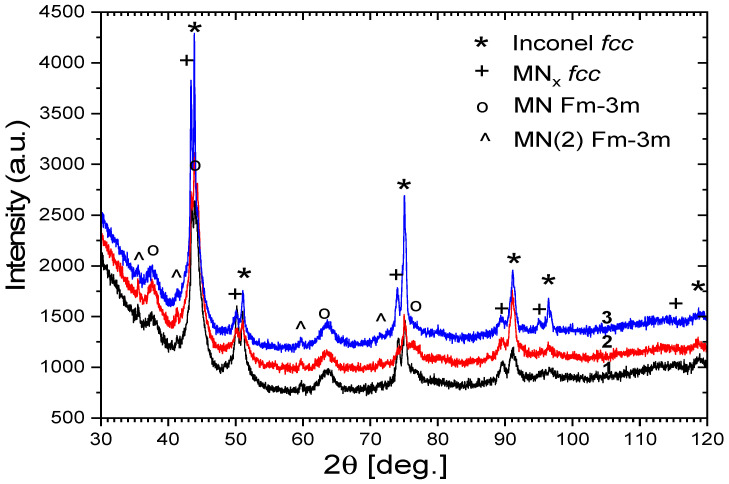
Diffraction patterns of samples after nitriding and HT-air-vibro machining in 3 different areas of the sample.

Figure 4 presents the diffraction pattern of the sample subjected to heat treatment in air (HT-air), vibro-machining, and subsequent nitriding. It should be emphasised that the nitriding of this particular sample was conducted under different conditions compared to the other specimens, which resulted in the formation of a discontinuous nitrided layer. Consequently, some differences in the diffraction results can be attributed to variations in the nitriding process parameters. Nevertheless, the data are included here for comparison and for a broader discussion of phase evolution.

The diffraction patterns presented in Figure 4, obtained for samples 1–3, reveal cubic phases with qualitatively similar phase compositions. The main phase is a metallic Inconel-type solid solution (Ni–Cr–Co) with an fcc structure and a lattice parameter of 3.574 Å. This phase appears to be slightly strained and elastically anisotropic, with subgrain structures of approximately 20–30 nm. The second identified phase corresponds to a solid solution of metals containing a small (several percent) amount of nitrogen, denoted as MN_x_; (fcc) (S-phase). This phase is considerably strained and anisotropically deformed compared to the primary metallic phase, with a lattice parameter of 3.609 Å and slightly smaller crystallites. The third phase is a metal nitride with the space group Fm-3m and a lattice parameter of about 4.14 Å, characterised by tiny crystallites (a few nanometres in size), consistent with CrN- or WN-type structures. The fourth phase, denoted as MN(2) (Fm-3m), exhibits a lattice parameter of approximately 4.37 Å and larger crystallites in the range of 20–30 nm. This phase occurs in smaller quantities compared to the previous nitride phase and corresponds to TaN-type structures.

### 3.3. Microhardness Analysis

**Figure 5:**
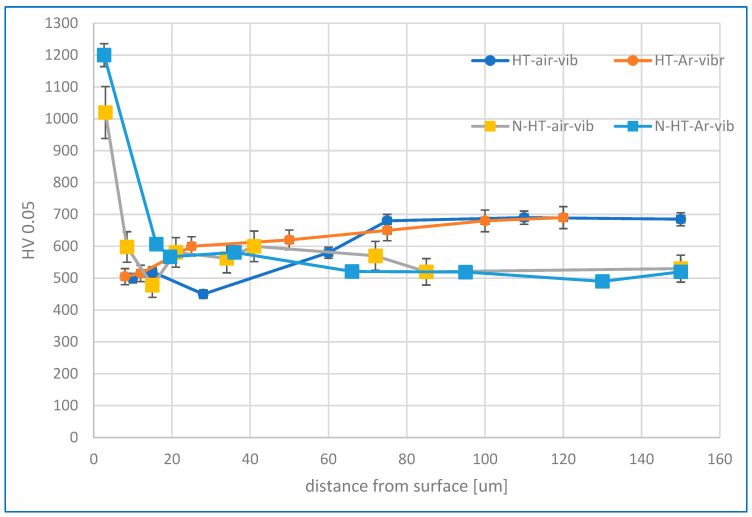
Microhardness of the sub-surface layer material after printing, heat treatment in air, argon, and abrasive machining and nitriding.

Figure 5 illustrates the changes in microhardness across the subsurface layer resulting from finishing treatments and the nitriding process. For samples subjected to both heat treatment variants followed by vibration machining, a reduction in microhardness was detected at the material surface within a layer approximately 30–50 µm thick. This surface softening can be attributed to different cooling conditions compared to the core material, which correlates with the previously observed microstructural variations and the phenomenon of microcracks appearing only in the sample interior while the surface layer remains defect-free.

The microhardness increased from approximately 500 HV0.05 at the surface to over 680 HV in the core for both heat treatment variants. However, samples heat-treated with air cooling exhibited a thicker softened layer (~30 µm) compared to argon-cooled samples, corresponding to the depth where oxide precipitates were observed. This hardness reduction may result from a decrease in Ti and Al concentrations in the near-surface zone due to diffusion toward the surface, with increased concentrations of these elements detected in the oxide precipitates.

The ASPN process significantly enhanced surface hardness for both heat treatment variants, although samples heat-treated in argon achieved higher surface hardness values (~1200 HV0.05). For nitrided HT-air samples, a hardness decrease was observed at the oxide precipitate boundary approximately 15 µm from the surface, similar to the pattern in mechanically treated samples. Notably, nitriding reduced the core material’s hardness to approximately 520 HV, compared to 680 HV in samples that received only heat treatment and vibro-abrasive processing. This core softening phenomenon may be attributed to the high temperature of the nitriding process, which could promote stress relief and microstructural changes in the bulk material.

**Figure 6:**
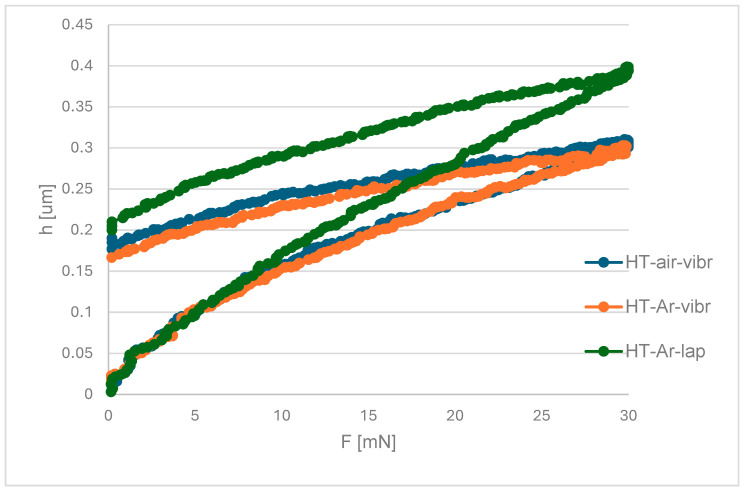
Indentation test for nitriding layers produced after different post-processing variants: heat treatment in air cooling, vibro machining and lapping (HT-air-vibr), heat treatment in Ar vibro machining and lapping (HT-Ar-vibr), and heat treatment in Ar and lapping (HT-Ar-lap).

Additionally, indentation tests were performed on metallographic microsections of the nitriding layer after vibro-machining and lapping, as well as two heat treatment variants. Young’s modulus, layer Vickers hardness, and the elastic part of indentation work ƞIT were determined. Average values and standard deviation are presented in Table 2, and the process progress is shown in Figure 6.

The obtained results indicate that the nitrided layer formed on HT-air samples exhibits slightly lower hardness and increased ductility compared to the HT-Ar variant. This behaviour is corroborated by microstructural analysis, which reveals chipping of the surface layer in HT-air samples (Figure 6), suggesting reduced cohesion and brittleness. For samples subjected to HT-Ar treatment followed by lapping only, the nitrided layer demonstrates significantly lower hardness and enhanced ductility. However, this effect may be partially attributed to the reduced layer thickness (3.8 ± 0.3 µm), where the substrate’s mechanical properties could influence the microhardness measurements due to the limited indentation volume relative to the layer depth.

The apparent lower hardness in lapped-only samples (HT-Ar-lap, 3.8 ± 0.3 μm) compared to vibration-treated samples (1200 HV_0.05_) reflects the substrate’s influence on indentation measurements. According to Johnson’s rule, measured hardness becomes unreliable when indentation depth (h_indent) exceeds 10% of coating thickness (t_layer). For the 3.8 μm nitrided layer with a 5 mN indentation load (resulting in h_indent ≈ 0.4 μm), the ratio h_indent/t_layer ≈ 0.11, near this critical threshold. When indentation depth reaches 10–15% of layer thickness, measured composite hardness is dominated by substrate properties (500–520 HV) rather than true coating hardness, producing artificially low readings. In contrast, thicker layers (6.4 μm) yield h_indent/t_layer ≈ 0.06, well below the threshold, ensuring measured hardness accurately reflects coating properties. Consequently, hardness values from lapped-only samples should be interpreted as composite hardness influenced significantly by the substrate rather than by the intrinsic nitrided layer hardness. The same criterion applies to Young’s modulus measurements: E values from thin-layer samples (3.8 μm) carry similar substrate artefacts and should be viewed as composite values, not layer-specific properties. For reliable property assessment, layers exceeding 6.0 μm thickness are recommended, where h_indent/t_layer < 0.10 maintains measurement reliability.

### 3.4. Scratch Test

Table 3 critical loads for nitriding layers for different variants of post-processing determined during scratch testing. Lc1 represents the applied load at which initial microcracking in the diffusion layer is detected; Lc2 represents the load at which adhesive failure initiates at the scratch track boundaries. HT-air samples exhibit higher Lc2 values due to stress buffering from the subsurface oxide gradient, while HT-Ar samples prioritise layer uniformity and thickness for thermal cycling durability.

**Figure 7:**
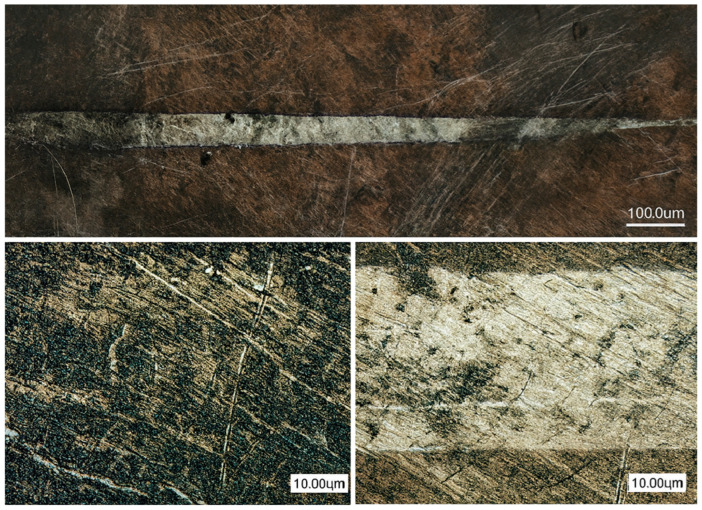
The lapping and nitriding surfaces after the scratch test for samples treated with air cooling and vibro-abrasive heat treatment.

**Figure 8:**
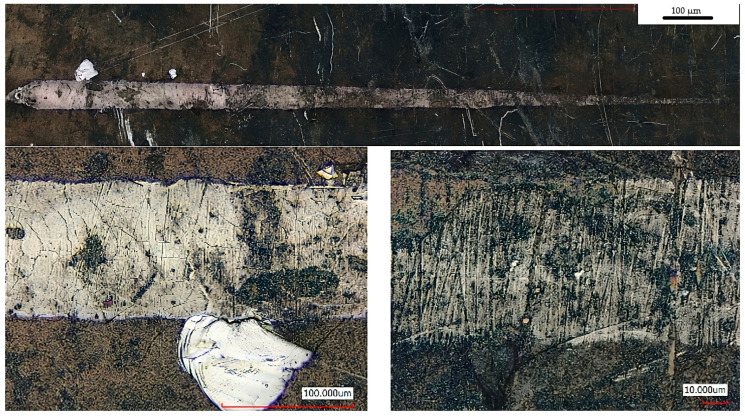
The lapping and nitriding surfaces after the scratch test for samples treated in argon (vibro-abrasive).

**Figure 9:**
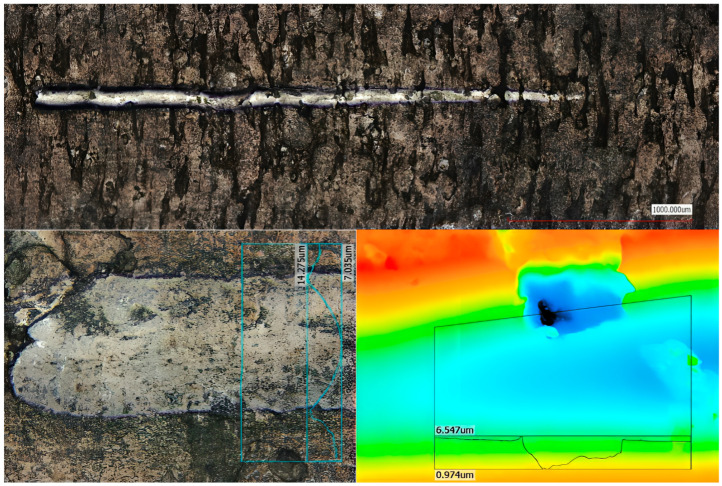
View of the surface after the scratch test for nitriding sample HT-Ar-vibr without lapping.

Figure 7, Figure 8 and Figure 9 shows the lapping and nitriding surfaces after the scratch test, respectively, for samples after heat treatment with air cooling vibro-abrasive (Figure 7), heat treatment in argon (vibro-abrasive) and nitriding (Figure 8), and heat treatment in argon and lapping (Figure 9). In critical locations, the surface is shown at higher magnification. Small cracks in the direction perpendicular to the indenter feed direction are visible for all variants post-processing. These cracks were recorded at a force of approximately 3N for the HT-air-vibro layer, while for the remaining samples, they were recorded at higher loads (Table 3).

Chipping at the scratch track borders occurred at similar critical loads across all tested samples (Table 3). The most durable nitrided layer was achieved following HT-air treatment with vibration and lapping, where initial chipping was recorded at approximately 18 N. Characteristic patterns at the scratch track boundaries were clearly visible for the HT-Ar-vibr sample (Figure 8), though complete spallation across the full width of the scratch track was not observed in any of the tested layers, indicating adequate adhesion strength.

Additional scratch testing was performed on a sample that underwent nitriding without subsequent lapping (HT-Ar-vibr), which exhibited the previously observed discontinuous, thin nitrided layer. Figure 9 presents the surface morphology following this test, where acoustic emission results remained comparable to those of lapped samples (Table 3). The layer cracks visible in the figure represent damage sustained at maximum applied load, demonstrating that despite the discontinuous nature of this thinner layer, its failure characteristics align with those of the more uniform, lapped variants.

These results suggest that while layer thickness and continuity influence absolute hardness values, the fundamental adhesion mechanisms and failure modes remain consistent across the different processing conditions, with the HT-air treatment providing optimal durability under scratch testing conditions. HT-Ar-vibr.

**Figure 10:**
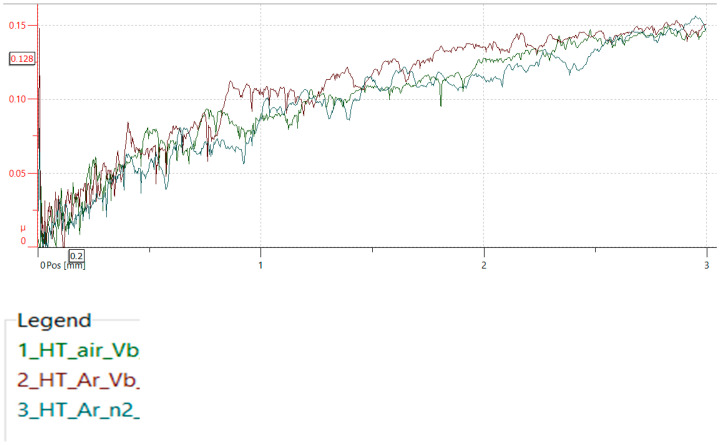
Friction coefficient for nitriding samples: 1- for HT-air vibro-machining (green), 2-HT-Ar (Red) and vibro-machining, 3-HT-Ar and lapping (blue).

The work also analysed changes in the friction coefficient as a function of load. Figure 10 shows the friction coefficient for nitriding samples: 1- for HT-air vibro-machining, 2-HT-Ar and vibro-machining, and 3-HT-Ar and lapping. As the load increases, the friction coefficient increases, reaching a maximum value of approximately 0.15 for a load of 30 N.

**Figure 11:**
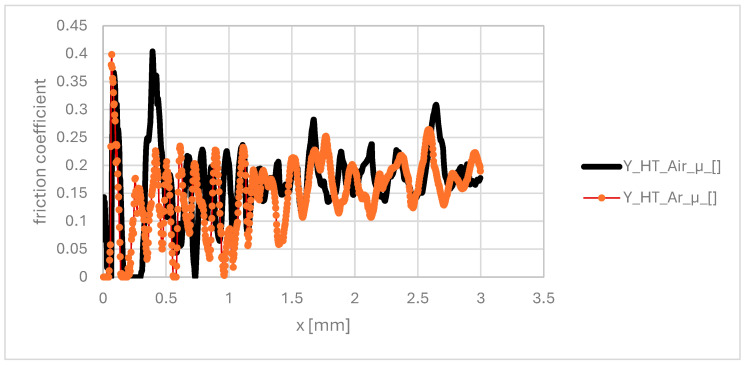
Friction coefficient for nitriding samples: 1- for HT-air vibro-machining, 2-HT-Ar and vibro-machining.

For samples nitrided without the lapping process, the friction coefficient changes are significantly greater, which is attributed to the higher surface roughness and the discontinuity of the nitrided layer (Figure 11).

## 4. Discussion

### 4.1. Heat Treatment Atmosphere: The Critical Role of Oxidation Control

The fundamental distinction between the two heat treatment routes emerges from oxidation thermodynamics during the cooling phase following secondary annealing. At 1073 K, both chromium and aluminium in the Inconel 939 matrix exhibit strong affinity for oxygen. When samples cool rapidly in atmospheric air, the surface layers preferentially oxidise due to steep thermal gradients that concentrate oxygen diffusion at the surface, combined with kinetic barriers that prevent oxygen redistribution into the bulk before the material cools below oxidation temperatures. This results in the continuous oxide layer observed in air-cooled samples (Figure 1a), consistent with findings by de Damborenea for LPBF Inconel 718 [45] and for other nickel-based superalloys.

The composition of this oxide layer—primarily Cr_2_O_3_ and Al_2_O_3_, as confirmed by EDS analysis (Figure 3c)—is critical for understanding subsequent nitriding behaviour. These oxides possess dramatically lower nitrogen diffusion coefficients than metallic Inconel: approximately 10^−16^ cm^2^/s in Cr_2_O_3_ compared to ~10^−10^ cm^2^/s in austenitic Ni-based matrices. This six-order-of-magnitude difference creates a severe kinetic barrier during the 450 °C nitriding process. Nitrogen must first dissolve at the oxide-plasma interface, then diffuse through the oxide layer before reaching the metallic substrate—a process that becomes rate-limiting and dramatically reduces nitrogen uptake. The result is precisely what we observe: EDS analysis reveals only 5–8 at.% nitrogen in air-cooled samples compared to 15–16 at.% in argon-cooled variants, directly reflecting this diffusion bottleneck.

In contrast, argon cooling suppresses oxidation by creating an inert atmosphere during the critical cooling window (1073 K → 700 K), where oxidation kinetics are fastest. While a thin surface oxide film (~0.5 μm) still forms through residual oxygen reactions, this minimal layer presents negligible diffusion resistance. Consequently, nitrogen penetrates rapidly and uniformly during ASPN at 450 °C, achieving diffusion depths of 6.4 ± 0.3 μm compared to only 3.8 ± 0.3 μm in air-cooled samples (Figure 2). This fundamental mechanism—oxide-controlled nitrogen transport—explains the ~40% difference in nitrided layer thickness and the pronounced hardness difference (1200 HV vs. 950 HV, Figure 5) despite identical nitriding parameters.

### 4.2. Surface Preparation: Competing Mechanisms and Optimisation Trade-Offs

The post-heat treatment surface preparation steps—vibratory finishing and lapping—do not simply polish surfaces; they initiate complex, competing mechanical and thermal effects that profoundly influence nitriding efficiency. Understanding these competing mechanisms reveals why the optimal sequence combines both treatments rather than using either alone.

Vibratory finishing with 6 mm steel media at 1800 rpm achieves multiple effects simultaneously. First, the mechanical polishing action reduces surface roughness from 5.49 μm (as-built LPBF) to 2.23 μm—a 2.5× reduction that eliminates asperities serving as stress concentrators. These surface asperities would otherwise channel localised nitrogen supersaturation during nitriding, triggering premature crack nucleation at their tips. The smoother surface distributes nitrogen uptake more uniformly, preventing the discontinuous layer formation observed in less-finished samples.

Second, the repeated impacts from steel media induce plastic deformation and generate dislocations at densities potentially exceeding 10^14^ cm^−2^ [46], as evidenced by the work hardening visible in microhardness profiles (hardness increases from 500 HV to 680 HV across the treated surface). These dislocations serve dual purposes: they act as short-circuit diffusion pathways for nitrogen penetration and as nucleation sites for uniform precipitation of nitride phases during ASPN. This dislocation effect accelerates nitrogen diffusion by 1–2 orders of magnitude through the dislocation pipe mechanism, directly explaining why vibro-abraded samples (6.4 μm layer) outperform lapped-only samples (3.8 μm, Figure 2), despite lapping producing smoother surfaces.

Third, vibratory finishing in air-cooled samples partially removes the continuous oxide layer, though residual oxygen-enriched zones remain visible in EDS maps (Figure 3). This removal exposes the fresh metallic surface to subsequent nitriding, improving local nitrogen uptake and layer formation in these regions.

However, subsequent lapping introduces a critical trade-off. While lapping further refines the surface to Ra 0.38 μm, eliminating remaining asperities and promoting ultimate layer continuity, the mechanical polishing action generates frictional heat (~100–200 °C locally) that anneal out the work-hardened layer created by vibratory finishing. This thermal annealing reduces the dislocation density that facilitates rapid nitrogen diffusion. Consequently, samples receiving only lapping (HT-Ar-lap) achieve thinner layers (3.8 ± 0.3 μm) despite adequate nitrogen availability—a counterintuitive result that reflects reduced diffusion efficiency from dislocation annealing.

The optimal sequence—vibration followed by lapping (HT-Ar-vibr-lap)—exploits this trade-off precisely: sufficient dislocation density from vibration remains after lapping to enable rapid diffusion, while surface quality is maximised. The result is the thickest and most uniform nitrided layer (6.4 ± 0.3 μm) with the highest hardness (1200 HV_0.05_). This demonstrates that surface preparation is not simply about roughness reduction; it is fundamentally about controlling the subsurface dislocation structure to enable efficient nitrogen diffusion.

The 10–15 μm oxide layer in air-cooled samples presents a diffusion barrier that cannot be substantially overcome by extending ASPN parameters. From first principles using Fick’s second law, the nitrogen flux through a diffusion barrier is as follows:

J = −D∂C∂x ≈ DΔCxJ = −D∂x∂C ≈ DxΔC

where J is the diffusion flux, D is the diffusion coefficient, ΔC is the concentration difference across the barrier, and x is the barrier thickness. The critical issue is the diffusion coefficient: nitrogen in Cr_2_O_3_/Al_2_O_3_ oxides is approximately D_N ≈ 10^−16^ cm^2^/s at 450 °C, versus D_N ≈ 10^−10^ cm^2^/s in austenitic Inconel. This six-order-of-magnitude difference makes the oxide layer rate-limiting regardless of temperature or time increases.

### 4.3. Mechanical Properties

The dramatic hardness increase following nitriding—from ~500–680 HV (post-HT/finishing) to 1200 HV (post-nitriding, HT-Ar)—results from two distinct but coupled strengthening mechanisms that operate simultaneously.

The primary hardening mechanism is lattice distortion. Nitrogen atoms occupy interstitial positions in the FCC lattice of austenite, expanding the lattice parameter from 3.574 Å (base Inconel, observed in XRD patterns) to 3.609 Å in the nitrided S-phase (Figure 4). This 1% expansion creates substantial compressive internal stresses within the nitrided layer, dramatically increasing yield strength. Hardness increase from lattice expansion is directly proportional to nitrogen content: the 15–16 at.% N in HT-Ar samples creates a significantly larger distortion than 5–8 at.% in HT-air samples, quantitatively explaining the 1200 vs. 950 HV difference (a 26% increase in hardness corresponding closely to the 2× difference in nitrogen content). This lattice distortion mechanism is well-established in the S-phase nitriding literature [47] and is the dominant hardening source.

Secondary hardening arises from precipitation of CrN and NbN nitride phases (Fm-3m structure, Figure 4) with crystallite sizes of a few nanometres. These precipitates provide dispersion hardening according to the relationship Δσ ∝ (f_v)^0.5^, where f_v is the volume fraction of precipitates. The higher nitrogen content in HT-Ar samples promoted greater CrN/NbN precipitation, further elevating hardness beyond the lattice distortion contribution alone. While secondary strengthening is quantitatively smaller than lattice distortion, it provides meaningful hardness augmentation.

The nanoindentation results provide additional evidence for the mechanical property differences. Elastic recovery values (nIT) of 49.5% for HT-Ar samples versus 38.5% for HT-air samples indicate superior toughness retention—less residual plastic deformation upon unloading reflects stronger, yet still ductile microstructures. This distinction proves critical: optical examination reveals chipping patterns (Figure 6) in HT-air samples suggesting brittleness, while HT-Ar samples maintain ductile characteristics. For thermal cycling applications where cyclic stresses are unavoidable, this ductility prevents crack initiation at layer-substrate interfaces—a subtle but crucial advantage for long-term performance.

The apparent lower hardness in lapped-only samples (HT-Ar-lap, 3.8 ± 0.3 μm) compared to vibration-treated samples warrants clarification. According to Johnson’s rule, indentation depth becomes problematic when it exceeds 10% of coating thickness. With 3.8 μm layers and ~0.4 μm indentation depth, this threshold is approached. When indentation penetrates to such depths relative to coating thickness, the underlying substrate (500–520 HV) begins to dominate the measured composite hardness, producing artificially low readings. This substrate effect does not indicate inferior layer quality—nitrogen incorporation and phase composition remain sound—but rather reflects a measurement artefact for thin coatings on soft substrates.

### 4.4. Adhesion Strength and Durability

Scratch testing reveals what initially appears to be a paradox: HT-air samples show superior durability under localised contact (Lc2 ≈ 18 N vs. 13.5 N for HT-Ar samples), yet HT-Ar samples are designated “optimal.” Understanding the mechanistic basis for this apparent contradiction requires examining the underlying stress states and failure mechanisms, then contextualising results within actual service requirements.

The superior scratch durability of HT-air samples stems directly from the oxide-enriched subsurface gradient zone (10–15 μm deep). This gradient structure functions as a compliant interlayer with lower elastic modulus than both the hard nitrided surface and the stiff metallic substrate. When the Rockwell C indenter (200 μm radius) progressively loads the surface, stress distribution follows a distinct sequence:

At initial contact (0–3 N), elastic deformation localises primarily in the hard nitrided surface layer. As loading continues (3–10 N), plastic flow initiates in the softer oxide gradient zone. Crucially, this intermediate-modulus zone redistributes stresses laterally, reducing localised peak stress concentration compared to what would occur if stress met the stiff metallic substrate directly. This stress-buffering effect delays crack propagation and enables the layer to withstand higher loads before adhesive failure. The phenomenon is well-documented in functionally graded coatings and multi-layer ceramic systems, where gradient structures provide superior impact resistance and durability under cyclic contact. It is worth noting that, for the HT-air-vibr-lap variant, the presence of this substantial oxide barrier hindered the creation of a continuous nitrided layer, whereas HT-Ar-vibr-lap proved significantly more effective, enabling robust S-phase formation which was comprehensively analysed. Given the large difference in nitrogen diffusivity in the oxide compared to the metal matrix, even extending nitriding time or increasing temperature nominally would result in only marginal improvement in HT-air samples under the current parameter window.

In contrast, HT-Ar samples lack this compliant oxide interlayer. The dense nitrided layer sits directly on the metallic substrate, with minimal intermediate stress distribution. The residual stresses from nitrogen lattice expansion (discussed in Section 4.3) create internal compressive stress (~1–2 GPa typical in S-phase layers). While beneficial for fatigue resistance—compressive stresses suppress tensile crack initiation—these stresses reduce the shear stress margin before adhesive failure, making the interface more susceptible to contact damage. Consequently, adhesive failure initiates earlier under scratch testing (Lc2 = 13.5 N).

However, this localised contact advantage for HT-air samples must be contextualised within intended service conditions for turbine blades. For such applications, scratch resistance from handling or assembly is a temporary concern; long-term thermal cycling durability is paramount. Under sustained high-temperature service (1000+ h at 700–900 °C under fatigue loading), gradient structures suffer critical disadvantages:

First, thermal cycling-induced stresses arise from differential expansion between the soft oxide layer, hard nitrided layer, and stiff metallic substrate. Each component has a distinct thermal expansion coefficient (CTE). Repeated thermal cycling creates cyclic shear stresses at internal interfaces—stresses that accumulate at the rate-limiting oxide-nitrided interface, eventually triggering delamination. This phenomenon is well-established in multi-layer and gradient coatings under thermal cycling [21,22].

Second, at service temperatures (700–900 °C), the oxide layer in HT-air samples oxidises further, forming increasingly thick Cr_2_O_3_ and Al_2_O_3_ precipitates that embrittle the interface. The initially beneficial stress-buffering oxide becomes a liability, promoting interface failure rather than preventing it.

Third, the thinner nitrided layer in HT-air samples (3.8 μm vs. 6.4 μm) provides insufficient protection depth for penetrating fatigue cracks that initiate from subsurface stress concentrators in the substrate. The thicker HT-Ar layer distributes fatigue stress over greater depth, preventing crack penetration to the substrate.

In contrast, HT-Ar samples’ thicker (6.4 ± 0.3 μm), uniform, denser nitrided layer can provide the following:

- Distributed fatigue stress over greater depth, suppressing subsurface crack initiation.
- Inherent oxidation resistance due to absence of an oxide layer boundary that could delaminate.
- Maintained adherence during thermal cycling because residual compressive stress counteracts tensile cycling stresses.

Long-term microstructural stability without embrittling interface oxidation.

Therefore, the “optimal sequence” designation for HT-Ar reflects deliberate application-specific optimisation. For turbine blade applications requiring sustained thermal cycling durability and creep resistance, accepting lower scratch resistance (13.5 N vs. 18 N) is a rational trade-off that prioritises long-term service life. This distinction is crucial: HT-air excels for components requiring short-term assembly/handling durability, while HT-Ar excels for aerospace turbine service. The choice represents engineering optimisation for specific use cases, not universal superiority.

### 4.5. Friction and Tribological Analysis

The friction coefficient results provide additional evidence for the robustness of the optimised post-processing sequence. For all lapped samples (HT-air-vibr-lap, HT-Ar-vibr-lap, HT-Ar-lap), the friction coefficient reaches approximately 0.15 at 30 N load, indicating consistent tribological performance regardless of the heat treatment atmosphere. This consistency arises because surface roughness dominates tribological behaviour: lapped surfaces (Ra 0.38 μm) essentially eliminate asperity contact, reducing friction to true sliding between hard surfaces according to μ ∝ (1/H) · (R_a), where hardness and roughness effects are decoupled.

Notably, the hardness difference between HT-air (950 HV) and HT-Ar (1200 HV) samples—26% higher in argon-treated variants—produced only ~8% variation in the friction coefficient, well within experimental scatter. This demonstrates that for optimally finished surfaces, tribological performance becomes independent of subsurface oxidation state, depending primarily on final surface quality.

In contrast, unlapped samples (HT-Ar-vibr, Ra 2.23 μm) exhibited significantly higher friction coefficient variability (Figure 11) due to asperity ploughing and material adhesion at contact spots. This emphasises that final lapping is not optional—it is essential for consistent tribological performance in service, confirming that the complete post-processing sequence (vibration + lapping) is required for industrial reliability.

The strong dependence of the friction coefficient on the surface finish rather than on the subsurface oxidation state has direct practical implications for component selection. Lapped samples (Ra 0.38 μm) consistently achieved low friction (μ ≈ 0.15) regardless of heat treatment variant, while unlapped samples (Ra 2.23 μm) exhibited higher, variable friction (μ ≈ 0.25–0.35). For components requiring low sliding friction—such as bearing interfaces, seal surfaces, or high-speed rotating elements where friction heat must be minimised—lapped variants are strongly recommended. Both HT-air-vibr-lap and HT-Ar-vibr-lap variants achieve acceptable friction performance; however, HT-Ar-vibr-lap is preferred due to superior hardness (1200 vs. 950 HV) and thermal cycling durability. Conversely, for wear-resistant applications involving abrasive contact (leading edge erosion, particulate impact), vibration-finished samples without lapping may be acceptable despite higher friction, as the slightly rougher surface (Ra 2.23 μm) provides mechanical interlocking with abrasive particles and the thicker nitrided layer (6.4 μm with higher hardness) compensates through extended wear-life. In summary, surface finish should be selected based on application priority: lapping for friction-sensitive applications and vibration-finish-only for abrasion-resistant designs.

### 4.6. Phase Formation and Microstructural Uniformity

XRD analysis confirmed formation of four distinct phases (Figure 4): base Inconel (FCC, a = 3.574 Å), expanded austenite S-phase γ’_N (FCC, a = 3.609 Å), CrN-type nitride phases (Fm-3m, a ≈ 4.14 Å with few-nanometre crystallites), and TaN/NbN-type phases (Fm-3m, a ≈ 4.37 Å with 20–30 nm crystallites). The phase composition variation between HT-air and HT-Ar samples reflects nitrogen availability rather than kinetic barriers during nitriding: high nitrogen concentration (15–16 at.%) in HT-Ar samples promoted extensive S-phase formation and higher CrN/NbN precipitation, while lower nitrogen (5–8 at.%) in HT-air samples limited phase formation, resulting in thinner, more discontinuous nitrided regions.

Surface preparation influenced phase uniformity: vibration-treated surfaces promoted uniform phase distribution across the nitrided depth through enhanced dislocation-mediated diffusion, while lapped-only surfaces showed local heterogeneity due to reduced dislocation density and uneven nitrogen uptake. The fine crystallite sizes (nm-scale) ensure high strength through Hall-Petch strengthening: σ_y = σ_0_ + (k/d^0.5^), where k = 0.5–1.0 MPa·m^0.5^ for nitride systems and d ≈ 5 nm. This grain refinement contributes an estimated 500–700 MPa strength increase, complementing lattice distortion hardening.

### 4.7. Process Implementation and Key Guidelines

The conducted investigation of post-processing parameters for LPBF Inconel 939 establishes a clear process pathway for achieving increased nitrided layer properties. The optimal sequence—argon heat treatment at 1073 K for 4 h, followed by vibratory finishing (4 h, 1800 rpm), final lapping to Ra < 0.4 μm, and plasma nitriding at 450 °C for 8 h in a 25% N_2_ + 75% H_2_ atmosphere—produces layers with thickness 6.4 ± 0.3 μm, surface hardness 1200 HV_0.05_, nitrogen content 15–16 at.%, and adequate adhesion strength (Lc2 ≈ 13.5 N). This sequence effectively eliminates the oxidation barrier that suppresses nitrogen diffusion in air-cooled samples, while the combined vibratory finishing and lapping steps optimise dislocation-mediated diffusion and promote uniform layer formation.

Heat Treatment Stage: Argon atmosphere during cooling is mandatory—air cooling introduces a 10–15 μm oxide layer that reduces nitrogen uptake by approximately 50%, resulting in thinner (3.8 vs. 6.4 μm), harder-to-process layers. If argon atmosphere is unavailable, preliminary studies indicate that controlled re-heating in argon at 200–400 °C for 30 min may partially recover nitrogen diffusion efficiency.

Surface Preparation Stage: Both vibratory finishing and lapping are essential. Vibratory finishing alone produces adequate surface quality (Ra 2.23 μm) but leaves residual work hardening that accelerates nitrogen diffusion. However, lapping without prior vibratory finishing reduces layer thickness by 33% due to annealing-out of beneficial dislocations. The combination of both treatments—vibration followed by final lapping—represents the optimal balance, producing the thickest and most uniform layers.

S-phase formation in γ′-strengthened Inconel 939 leads to expanded austenite and nitride precipitates at γ/γ′ interfaces. While these structures typically enhance room-temperature hardness and wear resistance, potential trade-offs (e.g., γ′ dissolution, nitride-induced embrittlement, altered creep/fatigue resistance at high temperature) must be acknowledged. As this work focuses on first-order mechanical and tribological assessment, extrapolations to long-term turbine conditions should be made with caution. Future studies should address the stability and performance of nitrided layers undeservice-like thermal cycling and high-temperature oxidation.

Nitriding Stage: The 450 °C temperature and 8 h duration used in this study delivered satisfactory layer thickness and mechanical properties. However, preliminary thermodynamic analysis suggests that lower temperatures (400–425 °C) on oxide-free (HT-Ar) surfaces may achieve similar layer properties with reduced thermal distortion, which would be particularly valuable for complex blade geometries. Nitriding duration beyond 8 h shows diminishing returns on layer thickness growth.

## 5. Conclusions

The research results demonstrate that both heat treatment and abrasive processing have a significant influence on the formation of nitrided layers on the surface of additively manufactured Inconel 939 components.

The most favourable properties of the nitrided layers—namely, high hardness, strong adhesion to the substrate, and a desirable phase composition—were obtained for samples subjected to heat treatment in an argon atmosphere, followed by vibro-abrasive machining and final lapping prior to plasma nitriding.

1. This study systematically investigated the influence of post-processing sequences, including heat treatment atmosphere (argon vs. air), vibratory finishing, and lapping, on the active screen plasma nitriding behaviour of additively manufactured Inconel 939. The key conclusions are as follows:
2. Impact of Heat Treatment Atmosphere

Heat treatment in an argon atmosphere (HT-Ar) effectively suppresses the formation of the thick Al/Cr-rich oxide layer present in air-cooled samples (HT-air). This oxide layer acts as a significant diffusion barrier, limiting nitrogen penetration during nitriding and resulting in thinner and discontinuous nitrided layers in HT-air samples (~3.8 µm), compared to uniform and continuous layers (~6.4 µm) achieved with HT-Ar.

3. Effects of Surface Preparation

The combination of vibratory finishing and final lapping optimises substrate surface conditions by reducing roughness and preserving beneficial dislocation densities. Vibratory finishing alone improves nitrogen diffusion through dislocation generation but leaves a rougher surface, resulting in higher friction coefficients. Final lapping produces smoother surfaces with lower friction but can reduce dislocation density if applied without prior vibratory finishing.

4. Nitrided Layer Properties and Trade-offs

The HT-Ar-vibr-lap process produces nitrided layers with significantly higher surface hardness (~1200 HV_0.05_) and more uniform S-phase formation, contributing to superior mechanical properties. However, scratch tests reveal that HT-air-vibr-lap samples have higher adhesion resistance under localised loading (Lc2 ≈ 18 N) due to the stress-buffering effect of the oxide gradient, which HT-Ar samples lack.

5. The findings suggest that HT-Ar-vibr-lap is optimal for applications requiring high hardness, wear resistance, and thermal cycling durability, such as turbine blades, due to its thicker, more uniform nitrided layers.

## Figures and Tables

**Table 1:** Chemical composition of Inconel 939 [18].

Element	Min	Max
Ni	Balance	Balance
Cr	20.0	23.0
Co	18.0	20.0
Ti	3.0	4.0
Al	1.5	2.0
W	1.0	3.0
Ta	1.0	2.0
Nb	—	1.2
C	—	0.15
Zr	—	0.15
B	—	0.015

**Table 2:** Mechanical properties of nitriding layers after two variants of heat treatment obtained from the nanoindentation test.

Nitriding Layer		Thickness [µm]	E [GPa]	HV IT	nIT [%]	HV 0.05	Lc1 [N]	Lc2 [N]
1-HT-air-Vibr	Mean	6.4	208.4	952	38.5	1012	2.999	17.997
	SD	0.33	3.9	159	5.6	112	0.35	2.03
2-HT-Ar-Vibr	Mean	6.4	183.8	1020	49.5	1200	12.745	13.495
	SD	1.1	29.4	98.7	1.7	116	1.52	1.80

**Table 3:** Critical loads for nitriding layers for different variants of post-processing determined during the scratch test.

Sample	Lc1 [N]	Lc2 [N]
HT-air-vibr-lap	2.999	17.997
HT-Ar-vibr-lap	12.745	13.495
HT-Ar-lap	7.498	12.742
HT-Ar-vibr	8. 992	11.988

## Data Availability

The original contributions presented in this study are included in the article. Further inquiries can be directed to the corresponding author.
